# IgA Nephropathy With a Membranoproliferative Glomerulonephritis Pattern Associated With Autoimmune Hepatitis: A Case Report

**DOI:** 10.7759/cureus.105937

**Published:** 2026-03-26

**Authors:** Hiroya Adachi, Haruna Noishiki, Hozue Ehara, Keiichi Tamagaki, Kazuhiro Sonomura

**Affiliations:** 1 Nephrology, Matsushita Memorial Hospital, Moriguchi, JPN; 2 Nephrology, Graduate School of Medical Science, Kyoto Prefectural University of Medicine, Kyoto, JPN

**Keywords:** autoimmune hepatitis, corticosteroid therapy, membranoproliferative glomerulonephritis, nephrotic syndrome, secondary iga nephropathy

## Abstract

Cirrhosis is a known cause of secondary IgA nephropathy (IgAN), often presenting with a membranoproliferative glomerulonephritis (MPGN) pattern and poor renal outcomes. We report a case of IgAN with an MPGN pattern associated with autoimmune hepatitis (AIH) in a 74-year-old woman. Although the patient had liver cirrhosis, her AIH was in clinical remission following prior corticosteroid therapy. She presented with nephrotic-range proteinuria and lower leg edema. Renal biopsy confirmed IgAN with an MPGN pattern. Notably, serum IgA levels were not significantly elevated, indicating that mechanisms beyond cirrhosis-related impaired IgA clearance were involved. Despite the typically poor prognosis associated with MPGN patterns, corticosteroid therapy led to rapid and sustained remission of proteinuria. This case suggests that immune dysregulation associated with AIH may play a primary pathogenic role in the development of aggressive IgAN phenotypes, independent of cirrhosis-related IgA accumulation.

## Introduction

IgA nephropathy (IgAN) is the most common form of primary glomerulonephritis worldwide; however, IgA deposition in the glomeruli can also occur secondary to various systemic conditions. While primary IgAN is an autoimmune disease driven by galactose-deficient IgA1, secondary IgAN is typically associated with underlying systemic diseases that alter IgA production or clearance. Chronic liver disease, particularly cirrhosis, is widely recognized as one of the most common underlying disorders associated with secondary IgAN. Glomerular IgA deposition is observed in up to 60.5% of patients with cirrhosis [1], and approximately 9% to 25% of these patients eventually develop clinical IgAN [2,3]. The pathogenesis has traditionally been attributed to increased intestinal IgA production and impaired hepatic clearance, leading to persistently elevated circulating IgA [4,5].

In some cases of hepatic IgAN, renal histology demonstrates a membranoproliferative glomerulonephritis (MPGN) pattern. This pattern typically reflects robust complement activation and severe immune complex-mediated injury. This specific histological variant is clinically significant, as it is frequently associated with nephrotic syndrome and carries a poor renal prognosis, with a high rate of progression to end-stage renal disease [2].

Autoimmune hepatitis (AIH) is a chronic inflammatory liver disease characterized by immune-mediated hepatocellular injury. Although IgAN is known to coexist with several autoimmune disorders, such as Sjögren’s syndrome and ankylosing spondylitis [6], reports describing the coexistence of AIH and IgAN are exceedingly rare, and their clinicopathological features remain poorly defined [7].

Here, we report a case of IgAN with an MPGN pattern that developed in a patient with AIH complicated by cirrhosis. Given the extreme rarity of this coexistence, the specific pathophysiological link remains unclear. This case addresses a significant knowledge gap by presenting an atypical occurrence of MPGN-pattern IgAN during the clinical remission of AIH, notably without elevated serum IgA levels, which showed an unexpectedly favorable response to corticosteroids. The clinical course suggests that immunological mechanisms related to AIH, rather than cirrhosis-associated IgA accumulation alone, might have contributed to the development of this specific renal phenotype.

## Case presentation

A 74-year-old woman presented to our hospital with progressive bilateral lower leg edema that had persisted for several weeks. She had a history of AIH, which was diagnosed five years prior, after the emergence of elevated serum transaminase levels (AST and ALT). At that time, laboratory evaluation revealed a significantly elevated antinuclear antibody titer (1:640), an elevated serum IgG level (2,315 mg/dL), and a positive anti-smooth muscle antibody (1:40). A liver biopsy indicated inflammatory cell infiltration within the hepatic parenchyma and hepatocellular eosinophilic necrosis, confirming the diagnosis of AIH. Oral prednisolone (PSL) therapy was initiated at a dosage of 35 mg/day, resulting in a favorable clinical response without subsequent relapses. The PSL dosage was gradually tapered to 5 mg/day over approximately one year and was discontinued six months prior to the current admission. During this five-year period, she subsequently developed liver cirrhosis during the disease course but remained clinically stable without biochemical evidence of active hepatitis. Additionally, the patient had donated a kidney to her daughter 12 years earlier. Importantly, prior to the current onset of edema, she had no history of proteinuria, hematuria, or decline in renal function. Her baseline serum creatinine level was 0.7 mg/dL (eGFR 62 mL/min/1.73 m²), and previous routine urinalyses were completely normal, indicating stable renal function following nephrectomy.

Upon admission, the patient was alert and oriented, with a body temperature of 36.5 °C, blood pressure of 142/58 mmHg, pulse rate of 63 beats per minute, height of 148.7 cm, and body weight of 51.7 kg. Physical examination revealed clear breath sounds without crackles, normal heart sounds without murmurs, and a flat, soft abdomen without tenderness. Pitting edema was present in both lower extremities. Detailed laboratory findings are summarized in Table 1. Urinalysis showed nephrotic-range proteinuria and microscopic hematuria. Blood tests revealed anemia, thrombocytopenia, and decreased estimated glomerular filtration rate (eGFR), whereas liver function tests (AST, ALT, and bilirubin) and serum electrolytes were within normal limits. Immunological evaluation showed a positive antinuclear antibody (ANA) titer, but other autoantibodies, including anti-smooth muscle antibody and ANCA, were negative. Serum IgA and complement levels were within normal ranges. Abdominal computed tomography revealed a liver with a dull margin and irregular surface, consistent with cirrhosis.

**Table 1:** Laboratory findings on admission Laboratory findings on admission showed nephrotic-range proteinuria and hypoalbuminemia despite normal liver transaminase levels. Bold typeface indicates abnormal values. eGFR: estimated glomerular filtration rate, IgG: immunoglobulin G, IgA: immunoglobulin A, IgM: immunoglobulin M, C3: complement component 3, C4: complement component 4, CH50: total complement activity, LKM-1: liver kidney microsome type 1, PR3-ANCA: proteinase 3 anti-neutrophil cytoplasmic antibody, MPO-ANCA: myeloperoxidase anti-neutrophil cytoplasmic antibody, RBC: red blood cell, HPF: high-power field.

Laboratory tests	Result	Normal range
Hematology		
Hemoglobin	9.7 g/dL	11.5–15.0
White blood cell count (WBC)	4.0 ×10³/μL	3.5–9.0
Platelet count	126 ×10³/μL	150–350
Renal and liver function		
Serum creatinine	1.0 mg/dL	0.46–0.79
eGFR	41.7 mL/min/1.73 m²	>60
Blood urea nitrogen (BUN)	19 mg/dL	8–20
Aspartate aminotransferase (AST)	28 U/L	13–30
Alanine aminotransferase (ALT)	12 U/L	7–23
Alkaline phosphatase (ALP)	72 U/L	38–113
γ-glutamyl transferase (γ-GT)	21 U/L	9–32
Total bilirubin	0.3 mg/dL	0.4–1.5
Serum proteins and inflammatory markers		
Total protein	6.2 g/dL	6.5–8.0
Albumin	2.9 g/dL	3.8–5.3
C-reactive protein (CRP)	0.14 mg/dL	<0.30
Immunology		
IgG	1,648 mg/dL	870–1,700
IgA	347 mg/dL	110–410
IgM	97 mg/dL	33–190
C3	109 mg/dL	73–138
C4	19 mg/dL	11–31
CH50	55.1 U/mL	30–53
Antinuclear antibody (ANA)	1:320	<1:40
Anti-mitochondrial antibody (AMA)	Negative	Negative
Anti-smooth muscle antibody (ASMA)	Negative	Negative
Anti-liver kidney microsome type 1 antibody (LKM-1)	Negative	Negative
PR3-ANCA	Negative	Negative
MPO-ANCA	Negative	Negative
Urinalysis		
Urinary protein	7.23 g/gCr	<0.15
Hematuria	5–9 RBCs/HPF	<5

**Figure 1:**
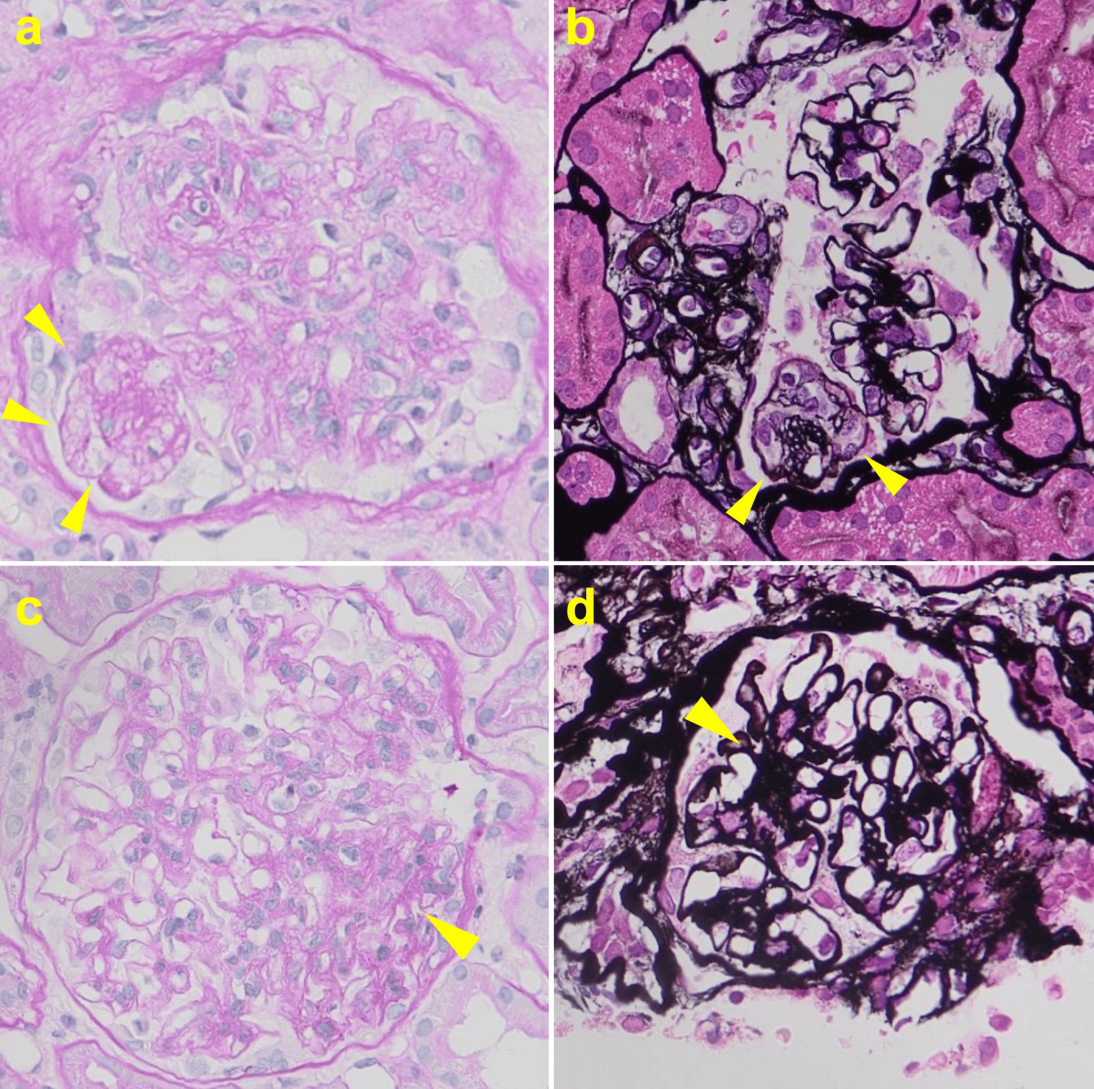
Light microscopic findings of the kidney biopsy (a, b) Mesangial lysis and duplication of the glomerular capillary loops (arrowheads); (a) periodic acid–Schiff staining, ×400; (b) periodic acid–methenamine silver staining, ×400; (c, d) mesangial cell proliferation (arrowheads); (c) periodic acid–Schiff staining, ×400; (d) periodic acid–methenamine silver staining, ×400.

**Figure 2:**
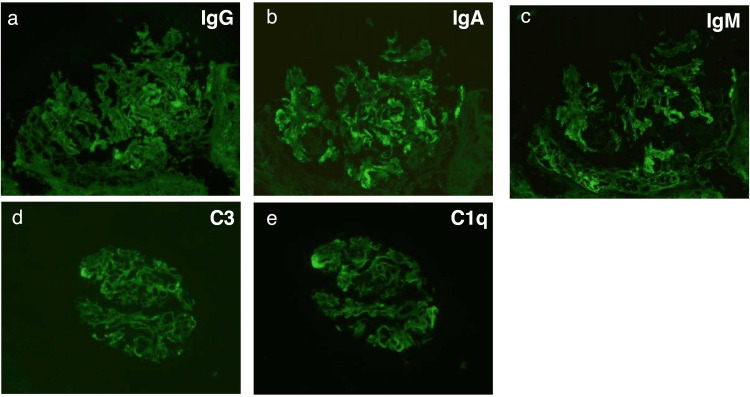
Immunofluorescence findings (a) IgG staining was negative; (b) IgA staining showed granular deposition (2+) in the mesangial areas and along the glomerular capillary walls; (c) IgM, (d) C3, and (e) C1q staining were weakly positive (±).

**Figure 3:**
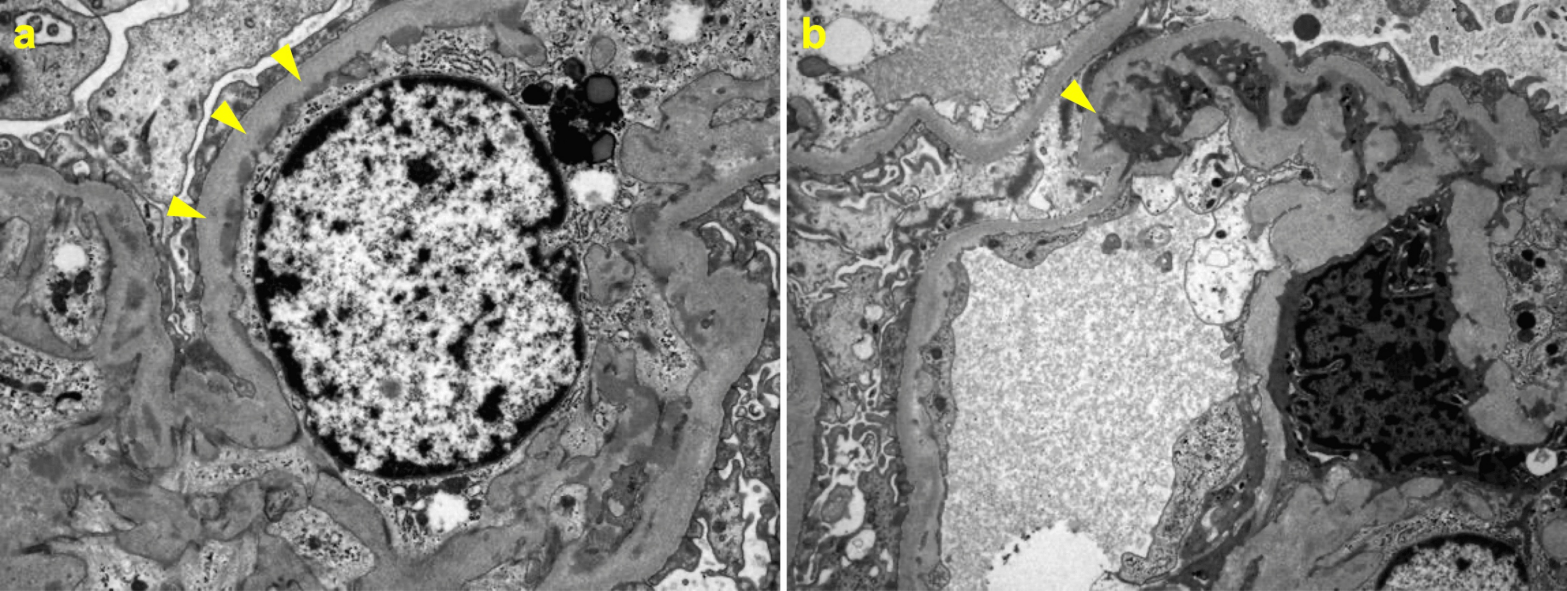
Electron microscopic findings (a) Electron-dense deposits in the mesangial area and perimesangium, accompanied by subendothelial deposits (arrowheads), (b) intermesangial electron-dense deposits (arrowheads).

A renal biopsy demonstrated global glomerulosclerosis in 11 of 22 glomeruli. The remaining glomeruli showed diffuse and segmental mesangial expansion with segmental mesangial cell proliferation. Mesangial lysis, accompanied by duplication of the glomerular basement membrane, was observed in two glomeruli (Figure 1). According to the Oxford classification, the MEST-C score was M1, E0, S0, T1, C0 (provided for descriptive purposes, acknowledging its primary validation in primary IgAN). Immunofluorescence microscopy demonstrated granular IgA deposition in the mesangial areas and along the capillary walls (Figure 2). Electron microscopy revealed electron-dense deposits in the mesangial and perimesangial regions, as well as subendothelial and intermesangial deposits (Figure 3). Based on these findings, a diagnosis of IgAN with an MPGN pattern was established.

**Figure 4:**
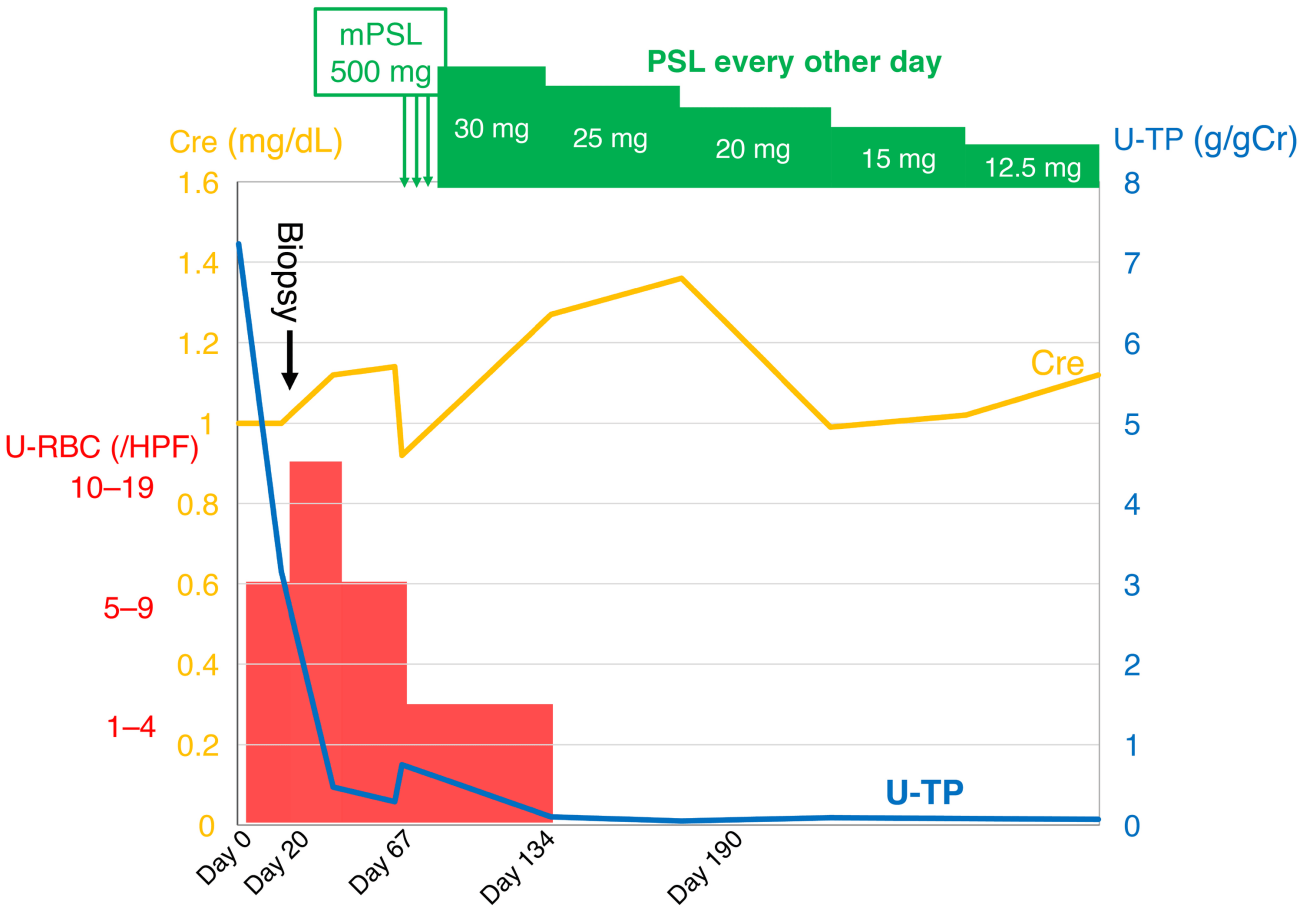
Clinical course of the patient Changes in urinary protein excretion (U-TP), serum creatinine (Cre), urinary red blood cell count (U-RBC), and methylprednisolone (mPSL) and prednisolone (PSL) dosage over time.

The patient was treated with intravenous methylprednisolone (500 mg/day) for three consecutive days, followed by oral prednisolone at a dosage of 30 mg every other day, with a planned taper of 5 mg every two months. Proteinuria markedly improved, leading to complete remission two months after the initiation of treatment (Figure 4). Remission has been maintained during follow-up.

## Discussion

Chronic liver disease, particularly cirrhosis, is recognized as the primary cause of secondary IgAN. Renal biopsies in patients with cirrhosis demonstrate glomerular abnormalities in up to 69.2% of cases, with IgA deposition observed in 60.5% using immunofluorescence microscopy [1]. Nine to 25% of patients with cirrhosis eventually develop IgAN [2,3]. The pathogenesis of hepatic IgAN has traditionally been attributed to increased IgA production and impaired hepatic clearance, resulting in persistently elevated circulating IgA [4,8]. Serum IgA concentrations in cases of cirrhosis-associated IgAN have been reported to be two to four times higher than normal [5].

In the present case, however, such a significant elevation of serum IgA was not observed at the onset of nephrotic syndrome. This finding suggests that mechanisms beyond cirrhosis-related IgA overproduction and impaired hepatic clearance might have contributed to the onset of the disease, raising the possibility that immune dysregulation associated with AIH, rather than cirrhosis alone, played a pathogenic role in the development of IgAN. Although distinguishing hepatic IgAN from primary IgAN based solely on histopathological findings is challenging, an MPGN pattern has been observed in approximately 20% of hepatic IgAN cases [2]. Moreover, patients with an MPGN pattern frequently develop nephrotic syndrome, and the renal prognosis is generally poor, with up to 44% requiring initiation of dialysis [2]. Despite the presence of an MPGN pattern in this case, urinary abnormalities improved rapidly following treatment, indicating a more favorable clinical course than typically reported.

To date, only a limited number of cases describing the coexistence of AIH and IgAN have been reported, and detailed clinicopathological characteristics are unclear [7]. The pathogenesis of IgAN is currently best explained by the widely accepted multi-hit hypothesis, which posits the following sequential processes: (Hit 1) an increased production of galactose-deficient IgA1 (Gd-IgA1); (Hit 2) the generation of autoantibodies, mainly IgG, directed against Gd-IgA1; (Hit 3) the formation of circulating immune complexes; and (Hit 4) mesangial deposition of these immune complexes, leading to subsequent glomerular inflammation [9].

Chronic inflammatory milieus associated with autoimmune diseases may directly drive this multi-hit process. In patients with AIH, elevated levels of B-cell activating factor (BAFF) have been reported both systemically and within the liver [10]. Excessive BAFF signaling promotes the survival and activation of autoreactive B cells, which may lead to increased production of autoantibodies against Gd-IgA1 (Hit 2) [11]. Furthermore, inflammatory cytokines such as interleukin-6 (IL-6), which are known to be elevated in AIH, have been shown to suppress the activity of IgA1 glycosylation enzymes, including C1GalT1, thereby directly promoting the production of Gd-IgA1 (Hit 1) [12]. Through these mechanisms, the immune dysregulation associated with AIH may simultaneously facilitate both the production of aberrantly glycosylated IgA1 and the autoantibody response against it.

Similar mechanisms have been proposed in other autoimmune diseases complicated by IgAN, such as Sjögren’s syndrome and rheumatoid arthritis, in which chronically inflamed tissues may serve as local sources of Gd-IgA1 and pathogenic antibodies [13,14]. In AIH, the liver may also serve as a reservoir for the production of aberrant IgA1 and autoantibodies, thereby contributing to the development of IgAN even in the absence of markedly elevated serum IgA.

In the present case, serum aminotransferase and IgG contents were within normal ranges at the onset of nephrotic syndrome, suggesting that AIH was in clinical remission. However, biochemical remission does not necessarily signify complete immunological quiescence. A histological hallmark of AIH is the dense infiltration of plasma cells within the portal tracts. A subset of plasma cells generated under chronic inflammatory conditions can persist as long-lived plasma cells, remaining resident within tissues and continuing to produce antibodies long after the resolution of overt inflammation [15]. Therefore, it is plausible that plasma cells activated during the active phase of AIH several years prior might have persisted in the liver and continued to secrete Gd-IgA1 or related autoantibodies, thereby perpetuating the pathogenic process that leads to IgAN.

It should be emphasized that the role of corticosteroid therapy in secondary IgAN is contentious, and immunosuppressive treatment is generally not recommended as standard therapy. The management of secondary IgAN focuses on addressing the underlying disease, with previous studies suggesting limited or inconsistent efficacy of corticosteroids in this context [6]. Accordingly, caution is warranted when interpreting treatment responses, and the findings in this case should not be generalized.

The favorable response to corticosteroid therapy observed in this case suggests that, in select patients, disease activity may be driven by immunologically active processes rather than solely by passive IgA accumulation. The therapeutic effects of corticosteroids in this case may be attributed to several mechanisms: (1) suppression of subclinical inflammatory cytokine signaling, particularly IL-6, leading to reduced de novo production of Gd-IgA1; (2) inhibition of BAFF/APRIL-dependent B-cell and plasma cell activation, resulting in decreased autoantibody production and immune complex formation; and (3) direct suppression of intraglomerular inflammation and complement activation. These effects are consistent with modulation of the IgAN multi-hit pathway and may explain the unexpectedly favorable response despite the presence of an MPGN pattern, which is typically associated with a poor renal prognosis.

Despite these observations, this report has several limitations. First, as a single case report, it cannot establish a definitive causal relationship between AIH and IgAN. The coexistence of these two conditions might be a coincidence. Second, we were unable to perform specific biomarker assays, such as immunostaining for galactose-deficient IgA1 (e.g., KM55 antibody), which could have further clarified the pathophysiological link. Therefore, our mechanistic inferences remain hypothesis-generating. Further accumulation of similar cases and basic research are required to elucidate the true relationship between autoimmune liver diseases and IgAN.

## Conclusions

This case suggests that immune dysregulation related to AIH, even during clinical remission, may contribute to the development and progression of IgAN. Although the possibility of IgAN arising independently cannot be entirely dismissed, the clinical course and treatment response indicate that AIH-associated immunological abnormalities might have played a pathogenic role in this case. Given the hypothesis-generating nature of this single case report, accumulation of similar cases and further mechanistic studies are warranted to elucidate the relationship between autoimmune liver disease and IgAN, as well as to optimize treatment strategies for secondary IgAN.

We report a case of AIH complicated by IgAN. The pathogenesis should be considered not only from the perspective of secondary IgAN associated with cirrhosis but also within the context of the coexistence of autoimmune disease and IgAN. When nephrotic-range proteinuria is observed in patients with AIH, the possibility of secondary IgAN with an MPGN pattern should be considered, and early renal biopsy may be warranted to inform appropriate therapeutic decision-making, including initiation of immunosuppressive therapy.
